# Deducing the presence of proteins and proteoforms in quantitative proteomics

**DOI:** 10.1038/s41467-018-04411-5

**Published:** 2018-06-13

**Authors:** Casimir Bamberger, Salvador Martínez-Bartolomé, Miranda Montgomery, Sandra Pankow, John D. Hulleman, Jeffery W. Kelly, John R. Yates

**Affiliations:** 1 Department of Chemical Physiology, The Scripps Research Institute, 10550 North Torrey Pines Road, La Jolla, CA 92037 USA; 2 Departments of Ophthalmology and Pharmacology, University of Texas Southwestern Medical Center, 5323 Harry Hines Boulevard, Dallas, TX 75390-9057 USA; 3 The Skaggs Institute for Chemical Biology, The Scripps Research Institute, La Jolla, CA 92037 USA

## Abstract

The human genome harbors just 20,000 genes suggesting that the variety of possible protein products per gene plays a significant role in generating functional diversity. In bottom-up proteomics peptides are mapped back to proteins and proteoforms to describe a proteome; however, accurate quantitation of proteoforms is challenging due to incomplete protein sequence coverage and mapping ambiguities. Here, we demonstrate that a new software tool called ProteinClusterQuant (PCQ) can be used to deduce the presence of proteoforms that would have otherwise been missed, as exemplified in a proteomic comparison of two fly species, *Drosophila* *melanogaster* and *D. virilis*. PCQ was used to identify reduced levels of serine/threonine protein kinases PKN1 and PKN4 in CFBE41o^−^ cells compared to HBE41o^−^ cells and to elucidate that shorter proteoforms of full-length caspase-4 and ephrin B receptor are differentially expressed. Thus, PCQ extends current analyses in quantitative proteomics and facilitates finding differentially regulated proteins and proteoforms.

## Abstract (web summary)

An accurate quantitation of different proteoforms remains challenging due to incomplete protein sequence coverage in mass spectrometry datasets. Here the authors describe a method to facilitate the discovery of proteoforms that may otherwise not be considered due to incomplete protein coverage or ambiguities in mapping peptides back to proteins.

## Introduction

Bottom-up proteomics employs the enzymatic digestion of proteins into peptides that are detected in the mass spectrometer, and subsequently identified and quantified using algorithms that search the data^1^. Regular advances in mass spectrometer technologies have led to better protein sequence coverage and have improved the depth of proteome coverage in bottom-up proteomic experiments^2^. Various software tools are able to map identified peptides back to proteins of origin, and report a list of proteins that explains all identified peptides^3–6^, taking into account that peptide sequences may be part of different proteins or proteoforms^7^. A standard proteomic experiment reveals the complexity of a proteome with sufficient fine resolution to detect proteins of almost all expressed genes as well as to differentiate transcriptional variants, splice variants, and post-translational modifications (aka, “proteoforms”) of individual genes^8–11^. However, proteomes often remain undersampled due to a large dynamic range in protein abundances and therefore bottom-up proteomics may currently be unable to comprehensively identify all proteoforms present in a proteome. One consequence of undersampling is that even when a bottom-up proteomics experiment is repeated precisely, the coverage of the proteome is similar but not identical. Highly abundant proteins are usually identified in both replicates, but detection of low abundant proteins and proteoforms is less reliable due to undersampling of low abundance proteoform-specific peptides.

This limitation restricts the ability to accurately measure changes in relative protein abundance between two experimental conditions. To minimize the effect of these inherent variations, proteomes can be labeled with different isotopes and compared in the same experiment^12^. While low abundance peptides are still undersampled, the use of isotope labels increases the comprehensiveness of relative peptide quantification in both samples. Therefore, while not a perfect solution, isotope labeling of samples can provide additional information on the differential abundance of proteoforms in either sample.

Currently, precise quantification of a proteoform is possible only when measured peptides are unique to a single proteoform^13^. This “Occam’s razor” approach^14^ avoids repeated use of peptides for quantification of disparate proteins by selecting the minimum set of proteins that explains the presence of the detected peptides. Alternatively, peptides can be grouped and an average relative abundance calculated based on protein FASTA annotations^15^ or by maximum parsimony per gene^16^. This approach reduces the repeated use of measurements to quantify different proteoforms, but it does not eliminate repeated use when quantifying proteins of different genes. Yet, another approach extrapolates a fraction of the measurement per proteoform that is dependent on the number of proteoforms known^17^. This approach runs the risk that the fractional ratio assignment may be skewed when additional proteoforms are present in the sample that were missed in the bottom-up proteomic experiment.

To identify differentially expressed proteoforms from quantitative proteomic datasets, all peptide-to-protein relationships derived from a bottom-up proteomics experiment are displayed in a bipartite network representation in which peptide nodes are connected to protein nodes and the edges between them indicate that the peptide sequence is part of the respective protein sequence. Proteins that share peptides belong to the same cluster and each cluster is one connected component in the network graph^18^. To construct a bipartite network from a set of peptide identifications, all corresponding proteins and proteoforms in a database (e.g., Uniprot^19^) are collected and the peptides and their corresponding proteins and proteoforms are assembled in a peptide-to-protein network in which peptide and protein nodes form two distinct sets. Moreover, quantitative information about both absolute and relative abundance is included in each peptide node. This kind of bipartite network allows a redundancy-free representation and interpretation of proteomic datasets and has previously been used to represent parts of a proteome. For example, Rinner et al.^20^ visualized peptide-to-protein clusters in Cytoscape^21^. However, a systematic analysis of peptide-to-protein networks for relative quantification of proteoforms in a two-sample comparison has not yet been realized.

Bipartite networks formalize the relationship between two sets of nodes and have been analyzed for quantitative traits based on weighted edges^22^ or for degrees of complexity, including hierarchy^23^. In proteomics, one set of nodes is typically based on experimental data (peptide identifications) and the second set is based on a pre-assembled database (all proteins encoded in a genome). Because a proteome is typically undersampled in a standard proteomics experiment, peptides may map to two or more discernable proteoforms listed in the database, leaving open the question of whether one or more proteoforms exist in the sample. However, in a quantitative proteomics experiment, the relationship between the two sets is not the only information available, as each peptide node is also associated with a quantitative measurement that reflects its relative presence in a two-sample comparison.

Here we introduce a new software tool called ProteinClusterQuant (PCQ) that exploits this quantitative information in bipartite peptide-to-protein networks to determine which proteoforms are differentially regulated or to infer the presence of additional, regulated proteoforms. We apply PCQ to two different proteomic datasets to demonstrate how to deduce the presence of proteoforms as well as to illustrate the limits of this in-depth proteomic data analysis. The first proteomic dataset comprises a comparison of the proteomes of two different fly species, whereas the second proteomic dataset comprises the proteomes of two isogenic cell lines that differ by a single point mutation. We show that PCQ detects significantly regulated proteoforms and can be used to deduce the presence of additional, regulated proteins or proteoforms that would have been missed otherwise.

## Results

### Comparing the proteome of two fruit fly species with PCQ

**Fig. 1:**
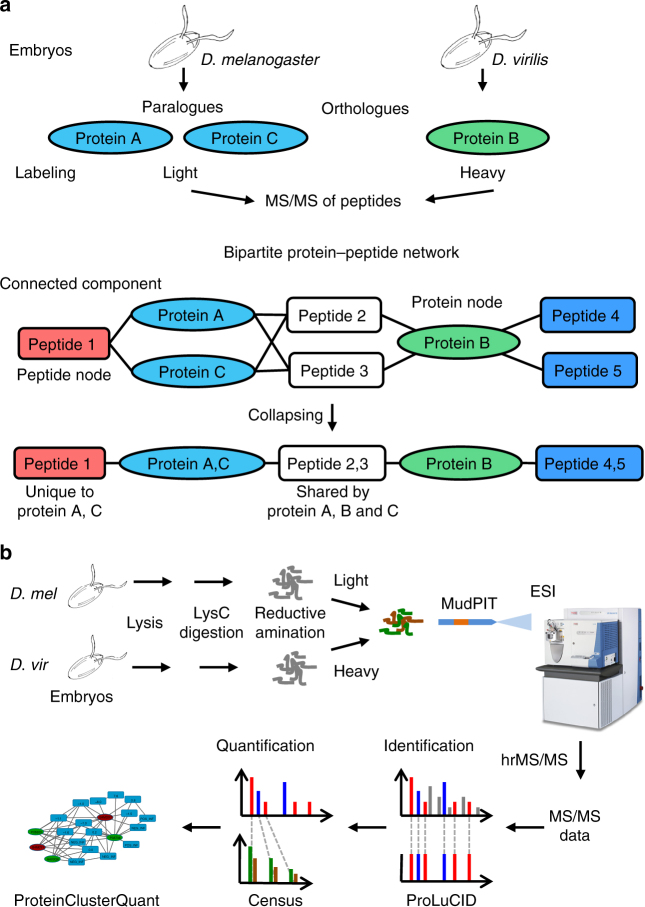
Peptide-to-protein clusters in bottom-up proteomics. **a** The schematic shows two orthologous proteins A or B (ellipses) present in *D*. *melanogaster* (blue) or *D*. *virilis* (green) embryos, respectively. Protein C is a paralog of protein A that is a result of a gene duplication in *D*. *melanogaster*. Proteins A and C are detected with a *D*. *melanogaster*-specific, unique peptide 1 (red rectangle) and protein B with peptides 4 and 5 (dark blue). Peptides 2 and 3 (white) are present in both orthologs A and B as well as in protein C. The peptide-to-protein cluster can be simplified in case proteins that share identical peptides are collapsed in one protein node and peptides that are shared by the same proteins are subsumed in one single peptide node. The *D*. *melanogaster* proteome is labeled light and *D*. *virilis* proteome is labeled heavy with isobaric isotopologues. **b** The schematic shows the workflow for a two-species comparison with isobaric isotopologue labeling. *Drosophila* embryos were lysed and digested with the endoprotease LysC, primary amines were dimethylated with isobaric isotopologues as light or heavy and the sample was analyzed with MudPIT on an Orbitrap series mass spectrometer. Peptides were identified with ProLuCID in a database search and isobaric isotopologues subsequently quantified with Census and peptide-to-protein networks analyzed in ProteinClusterQuant. Abbreviations: *vir*: *virilis*, *mel*: *melanogaster*, ESI: electrospray ionization, MS: mass spectrum

To test whether PCQ can be used to deduce the presence of additional, regulated proteoforms in a proteomic dataset, we set up an experiment that compared the proteome of two fruit fly species, *Drosophila* *melanogaster* and *D.* *virilis* (Fig. 1a). *Drosophila* *melanogaster* belongs to the subgenus *Sophophora*, whereas *D*. *virilis* belongs to the subgenus *Drosophila*. These two fruit fly species are ~40–60 million evolutionary years distant from each other^24^, so their proteomes are similar, but not identical. Genes that are present in both species represent orthologs and a pair of orthologous proteins may have identical amino acid sequences in evolutionarily conserved segments while differing in less conserved segments. In a bipartite peptide-to-protein network, orthologs are separated into two different protein nodes that are connected by shared peptides and differentiated by peptide nodes that include peptides unique to each ortholog (Fig. 1a). By exploiting the species specificity of these peptide sequences we were able to assess whether the method can generally be used to deduce the presence of proteoforms in a quantitative proteomics dataset.

Embryos (0 to 6 h old) of each *Drosophila* species were collected and lysed, and the proteins were precipitated and digested into peptides. The primary amines (lysine and N terminus) in the peptides were dimethylated with a distinct isotope-defined formaldehyde and sodium cyanoborohydride for each species^25^. In this proof-of-principle experiment, species were labeled either light (*D*. *melanogaster*) or heavy (*D*. *virilis*). Peptides of six independent replicate experiments were each chromatographically separated and detected by mass spectrometry with an Orbitrap Fusion (Thermo). Using a minimal length of six amino acids, mass spectra were searched for peptide identifications with Prolucid^26^, which is based on the SEQUEST search algorithm^1^, and peptide spectrum matches (PSMs) were filtered with DTASelect2^3^ to a false discovery rate (FDR) ≤0.14% (Fig. 1b, Methods). This proteomic dataset represented an experiment in which orthologs were most likely to be present in either sample, but may have been missed due to undersampling.

To thoroughly analyze the presence of orthologs in this dataset, we utilized PCQ to automatically perform an interference-free quantification of isobaric isotopologue-labeled peptides^27^ according to a user-adjustable set of parameters (Supplementary Data 1). To compare the two *Drosophila* species, PCQ generated a bipartite peptide-to-protein network and implemented SanXoT^28^ to accurately quantify proteins and proteoforms based on the relative isotope ratio measurements extracted from mass spectrometric data with Census^29^ (Supplementary Methods). PCQ mapped 1308 quantified peptides to 803 proteins and grouped these in 373 peptide-to-protein clusters (“Network 1”, Supplementary Data 2). The size of the bipartite network depends on the settings chosen during peptide identification. For example, increasing the FDR threshold for peptide identifications increases the size of the network, primarily through additional, single peptide-to-protein clusters. The output of PCQ conveniently represents the proteome as a bipartite peptide-to-protein network that also reports the difference in abundance of a peptide node in the two samples.

**Fig. 2:**
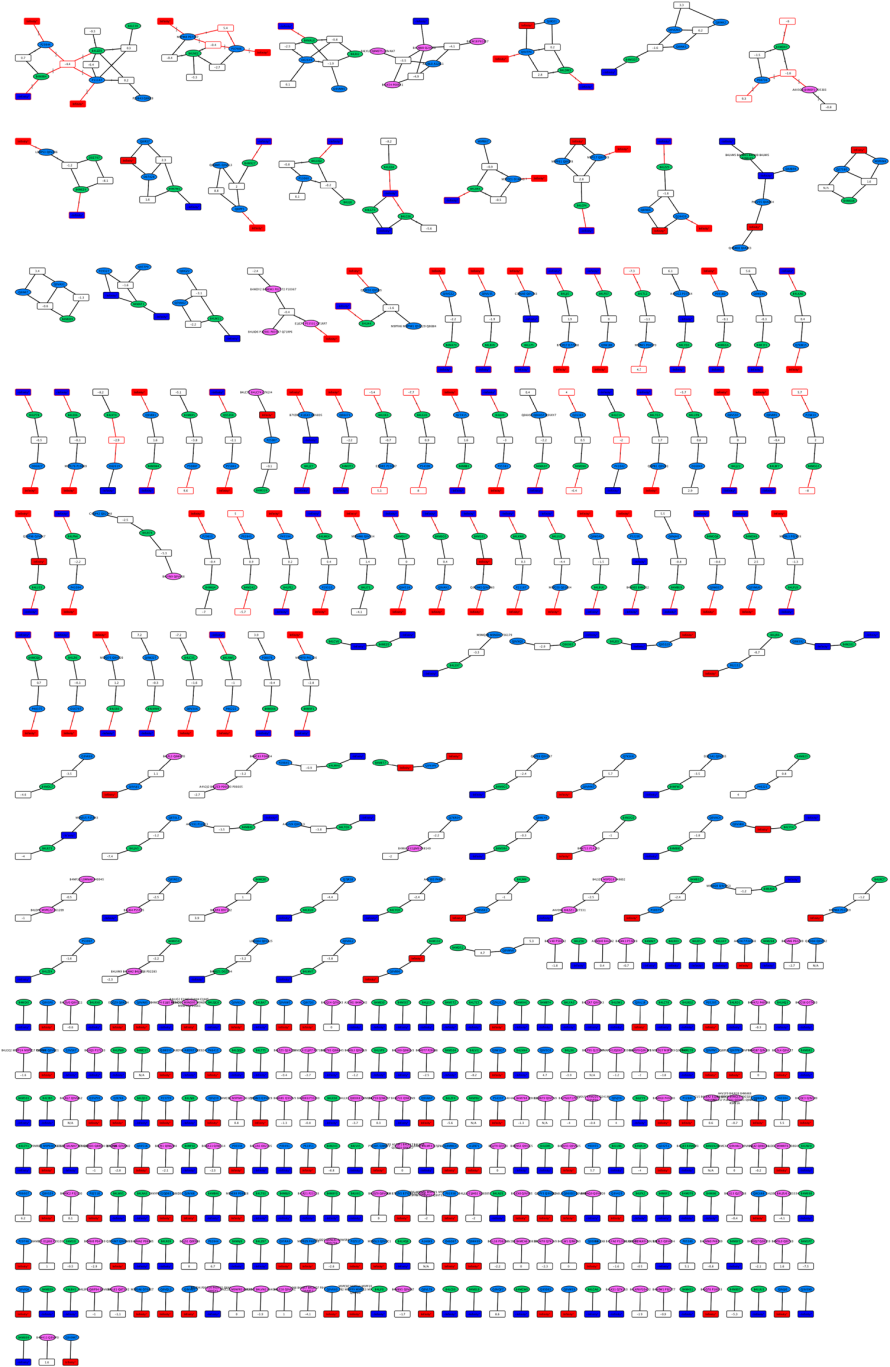
Complete peptide-to-protein network in the *D*. *melanogaster* vs. *D*. *virilis* species comparison. The network shows redundant peptides or proteins in single nodes. Peptide nodes are displayed as rectangles and protein nodes are ellipses (blue for *D*. *melanogaster*, green for *D*. *virilis*). Protein nodes comprising proteoforms of both species are shown in pink. Edges and peptide node outlines in red indicate that the relative quantification significantly deviates from the additional peptide nodes in a protein pair within a peptide-to-protein cluster. Relative abundance of peptide nodes is in the two-sample comparison is color-coded: peptide nodes in white, red, or blue are measured with a ratio, +∞ (+INF), or −∞ (−INF), respectively. The network is available online: Network 2,

To simplify the network, PCQ can subsume peptides that share the same connectivity into a single peptide node (Fig. 1a). Likewise, proteins that share the same connectivity are condensed into a single protein node that encompasses two or more different proteins or proteoforms. This step reduced the size of the bipartite network from 1308 to 597 peptide nodes and from 803 to 525 protein nodes (Fig. 2, “Network 2”, Supplementary Data 3). The resulting bipartite network is a non-redundant and complete representation of the identified and quantified proteome. Notably, the bipartite network included protein nodes that were not confirmed by the presence of a uniquely associated peptide node, leaving open the question of whether they were indeed present.

### Orthologous proteins and species specificity

**Fig. 3:**
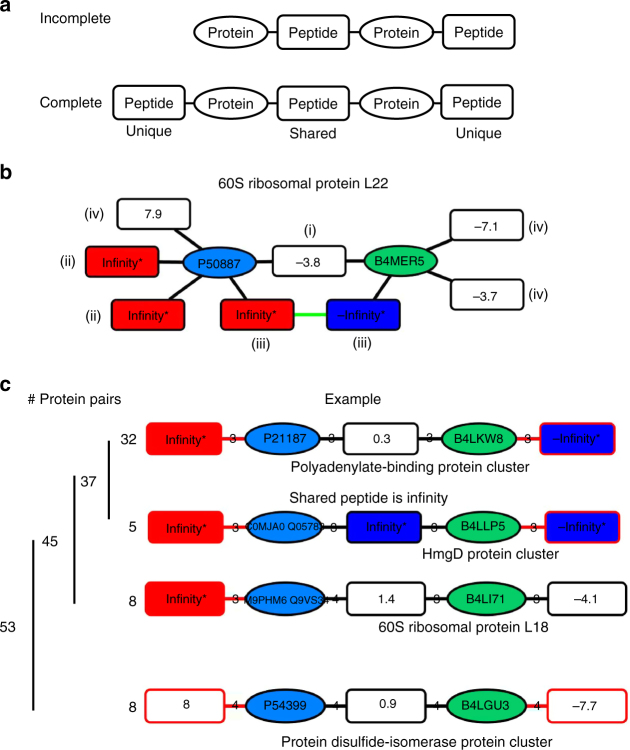
Quantification of relative protein abundance in a peptide-to-protein cluster and classification of complete protein pairs based on ratio measurements. **a** Schematic of an incomplete and a complete protein pair. Peptide nodes are displayed as rectangles and protein nodes are ellipses. **b** The peptide-to-protein cluster of the *D*. *melanogaster* (P50887, blue ellipse) and *D*. *virilis* (B4MER5, green ellipse) orthologs of 60S ribosomal protein L22 (RpL22) is depicted. The relative peptide abundance (Rc) is indicated in each peptide node: (i) denotes peptides that are shared by two different protein nodes; (ii) and (iii) highlight peptides that are present exclusively in one sample (unique peptide nodes). The expected ratio value is *n*:0 or log_2_(*n*/0) = + ∞ for a *D*. *melanogaster*-specific peptide (light isotope label, red rectangles) or 0:*n* or log_2_(0/*n*) = −∞ for a *D. virilis*-specific peptide (heavy isotope labels, green rectangles). The two-species-specific peptides (iii) are connected by an additional edge in green to indicate ≥80% sequence similarity (Supplementary Methods and Note 7). Species-specific peptides that were measured with a ratio value, although an infinity value is expected, are indicted with (iv). **c** Four different groups of protein pairs are shown. Each group subsumes protein pairs with similar ratio values for the unique as well as shared peptide nodes. Edges and nodes rendered in red indicate that this peptide node is significantly regulated within the protein pair (protein pair-centric analysis, see below). The number of protein pairs identified for each classification is indicated on the left as well as a brief description for each group is given in italic. One example for each group is shown

Confirmation of the presence of a proteoform currently depends on whether it was detected and identified with a unique peptide node. If a first protein node was identified only by peptides that are shared with a second protein node, direct evidence for the presence of the first protein node is missing (Fig. 3a). The two protein nodes thus constitute a pair of proteins with incomplete peptide information or an "incomplete protein pair" because a peptide node that is unique to the first protein node is absent. In contrast, a "complete protein pair" includes one peptide node that is shared by both protein nodes and two additional peptide nodes that are unique to either protein node. Therefore, all protein pairs in a bipartite network can be classified as either complete or incomplete, depending on whether both protein nodes associate with at least one unique peptide node or not.

Next, we evaluated if quantification of peptide nodes matched expectations in orthologous protein pairs. Orthologs can constitute a protein pair in case they have at least one peptide in common. The proteomic dataset included 53 complete protein pairs of orthologs. Figure 3b shows the ribosomal protein-like 22 (RpL22) peptide-to-protein cluster as example. The two RpL22 orthologs are separated into two protein nodes that are connected by a peptide node that subsumes all peptide sequences that were identified and are identical between the two orthologs. Because peptides were differentially isotope labeled according to species specificity, the ratio value of the shared peptide node (i) in Fig. 3b reflected the relative difference in abundance of the orthologs. The *D*. *virilis* RpL22 ortholog was >10-fold more abundant in *D*. *virilis* embryos than its *D*. *melanogaster* ortholog in *D*. *melanogaster* embryos.

In case peptide nodes are unique to a species-specific ortholog, they are present in only one of the two samples and thus are expected to be exclusively light or heavy labeled, which is infinity upon logarithmic conversion. Overall, infinity values were measured for at least one unique peptide node in 45 of the 53 complete protein pairs in the experiment (Fig. 3c), and in 37 of these, infinity ratio values were measured for both unique peptides, thus indicating the correct species specificity. In eight protein pairs infinity ratio values were measured for only one of the two unique peptide nodes, whereas the second unique peptide node was measured unexpectedly with a ratio value instead of infinity. This unexpected ratio value suggested that the peptide was present in both samples and thus in both *Drosophila* species even though it was reported in only one of the two-species-specific protein databases. Moreover, both unique peptide nodes were unexpectedly measured as present in both species in 8 of the 53 complete protein pairs. For example, in the RpL22 peptide-to-protein cluster mentioned above, ratio values for peptides that were unique to one ortholog only suggested incorrectly their presence in both samples and therefore species ((iv) in Fig. 3b). Overall, quantification did not match the peptide’s species specificity in 24 out of 106 unique peptide nodes (22.6%). This high proportion of mismatches compelled us to assess possible experimental errors that potentially limit quantification accuracy.

### Chemical noise limits quantification accuracy

We systematically analyzed all orthologs detected in the experiment and found that 326 *D*. *melanogaster*- and 411 *D*. *virilis*-specific peptide nodes were quantified correctly as solely present in one species (Table 1). Fifty-nine (13.8%) of *D*. *melanogaster*-specific and 44 (9.7%) of *D*. *virilis*-specific peptide nodes were incorrectly quantified as present in both species (103 out of 358 peptides with ratio measurements).

**Table 1:** Species specificity and quantification result of peptides

Measurement	Species	# of peptides	Comment
R	*D*. *mel* and *D*. *vir*	358	Expected
+∞ or −∞	*D*. *mel* and *D*. *vir*	98	Possible
+∞	*D*. *mel*	326	Expected
−∞	*D*. *mel*	6	Incorrect
R	*D*. *mel*	53	Incorrect
+∞	*D*. *vir*	0	Incorrect
−∞	*D*. *vir*	411	Expected
R	*D*. *vir*	44	Incorrect
		0	Discarded

The table shows the number of peptides that were identified and quantified depending on quantitative measurement (ratio value *R*, log_2_-transformed) and species specificity with no ion count threshold (Supplementary Data 1)

**Fig. 4:**
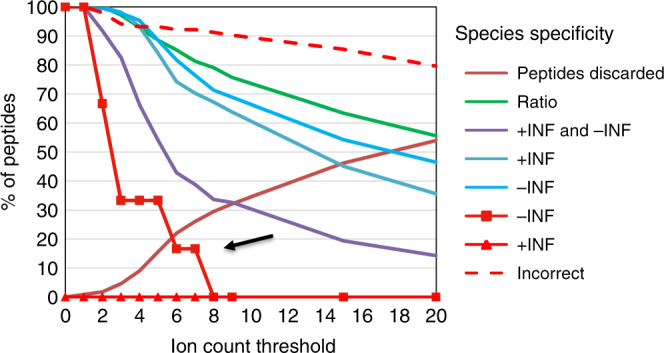
The number of quantified peptides decreases with increased ion count threshold per peptide node. The graph shows the percentage of peptides retained in dependence of a minimum number of ion counts per peptide node. The relative number of species-specific peptides that are quantified as expected, possible, or incorrect, dropped with increasing threshold for the number of ions identified. Note that the relative number of peptides with incorrect quantifications (e.g., *D*. *virilis*, +∞ and *D*. *melanogaster*, −∞) dropped more sharply than peptides measured with expected ratios. The black arrow points to a peptide which is not in the database version used (UniprotKB/TrEMBL release 2014_05)

One source of error we identified was chemical noise introduced randomly during mass spectrometric data acquisition (Supplementary Note 1). Chemical noise most likely accounted for most of the measurement errors because ratio values were strongly biased towards the correct species specificity (53 of 59 *D*. *melanogaster*-specific and all 44 *D*. *virilis*-specific peptides). However, six *D*. *melanogaster*-specific peptides were quantified with a negative rather than positive infinity value, incorrectly suggesting their presence solely in *D*. *virilis*. When we increased stringency by filtering the dataset to ≥3 fragment ion measurements per peptide node (Supplementary Data 1), 5 of the 6 incorrectly quantified peptides were eliminated, while >97% of peptide nodes were retained (Fig. 4, “Network 3, Supplementary Data 4). Thus, imposing more stringent requirements for quantification filtered out random noise so that only 1 out of 277 (0.4%) species-specific peptide nodes remained assigned to the incorrect species.

### Deducing the presence of orthologs

After filtering for chemical noise, we addressed the question of whether orthologs were missed during data acquisition and analysis. Thirty-eight protein pairs had one protein node that was not validated by a unique peptide node (and thus, these protein pairs were incomplete). If the two peptide nodes, which were present in the incomplete protein pair, differed markedly in ratio values (∆), it indicated that the ortholog might still be present despite the absence of a peptide node that is unique to it. Fourteen out of 38 incomplete protein pairs had very similar ratio measurements or identical infinity for the two peptide nodes which did not further support that the protein node in question was present. Twenty-four out of 38 incomplete protein pairs had an infinity measurement for the unique peptide node and a ratio measurement (or infinity of opposite sign) for the shared peptide node and therefore displayed a ratio discrepancy. This discrepancy in ratio values between the two peptide nodes suggested that the not yet further  validated protein node was present in either one or both samples.

**Fig. 5:**
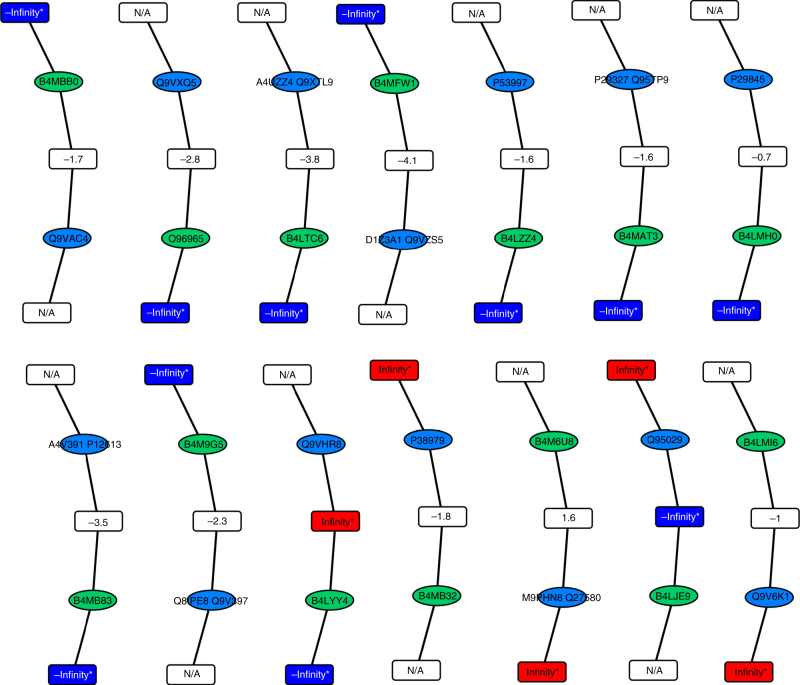
Fourteen incomplete protein pairs were converted in complete protein pairs upon considering identified but non-quantified peptide nodes. Protein pairs of the *D*. *melanogaster* (blue ellipses) and *D*. *virilis* (green ellipses) protein nodes are shown. The relative peptide abundance (Rc) is indicated in each peptide node. A value indicates a relative log_2_(Rc) abundance in both species, +Infinity (red rectangles) its presence in only *D*. *melanogaster*, –Infinity (green rectangles) its presence in only *D. virilis*, and “N/A” that the peptide node was solely identified but not quantified in its relative abundance

Seeking experimental evidence for the presence of these protein nodes, we re-analyzed the dataset. In this analysis, we included peptides that were identified but not quantified. Adding non-quantified peptide nodes to the peptide-to-protein network increased the size of the network (“Network 4”, Supplementary Data 5) and converted 3 (21%) incomplete protein pairs with similar ratio values into complete protein pairs (Supplementary Note 2) and, more importantly, it complemented 14 (58%) of the incomplete protein pairs with discrepant ratio values (Fig. 5). Thus, the presence of 10 additional *D*. *melanogaster*-specific and 4 *D*. *virilis*-specific protein nodes was not only supported by markedly different peptide ratio values within the protein pair but was also confirmed by peptide nodes that were unique to the respective protein node but were not quantified. The higher rate of conversion of incomplete into complete protein pairs in case they have discrepant rather than similar ratio values (58 vs. 21%) indicated that a difference in ratio values indeed supported the presence of a protein node even in the absence of a unique peptide node.

### Comparison of two isogenic cell lines with PCQ

To illustrate how the presence of proteoforms can be deduced in a complex human proteome with PCQ, we compared two human bronchial epithelial cell lines with quantitative proteomics. The cell line CFBE41o^–^ expresses a proteoform of the Cystic Fibrosis Transmembrane Conductance Regulator (CFTR) with a deletion of phenylalanine 508 (∆F508 CFTR) which is the underlying cause of the genetic disease Cystic Fibrosis in over 70% of patients. The control cell line HBE41o^–^ expresses wild-type (wt) CFTR and is isogenic to CFBE41o^–^. Thus, any change in protein expression is most likely a consequence of the phenylalanine F508 deletion in the ∆F508 CFTR protein. CFBE41o^–^ and HBE41o^–^ cells were labeled with light and heavy isobaric isotopologues, respectively, and analyzed in a single experiment (*n* = 1)^27^. The peptide-to-protein network was filtered with a threshold of at least three isobaric isotopologue peaks per peptide node. Seven thousand one hundred and fifty-seven peptide nodes and 7600 protein nodes assembled into 3965 connected components with a subset of peptide nodes that were significantly enriched in one of the isogenic cell lines (Supplementary Methods, “Network 5”, Supplementary Data 6) or present in only one of the two cell lines like the peptide-to-protein cluster comprising different LMO7 proteoforms (Supplementary Note 3). Overall, the bipartite network included 1564 out of 3965 connected components that contained more than one protein node.

Having assembled the bipartite peptide-to-protein network, we next determined whether any incomplete protein pairs with discrepant ratio values were present. Eighty-six out of 712 (12%) incomplete protein pairs showed a ∆ > 2-fold (~1*σ*) difference in ratio values in constituent peptide nodes. The difference in ratio values suggested that the additional protein node was not only present but also differed in relative abundance, although it was not identified with a unique peptide node (Supplementary Note 4). Notably, these differentially regulated proteoforms were often shorter cleavage products of a longer proteoform. Protein products that arise from endogenous enzymatic processing during maturation in vivo can be difficult to distinguish from their full-length precursor in bottom-up proteomics because a mature protein product might differ by only one peptide from the full-length precursor following endoproteolytic digestion in vitro. Therefore, protein products of endogenous proteolytic activity in vivo are very likely detected as a non-validated protein node in an incomplete protein pair.

For example, caspase-4 in its full-length pro-enzymatic proteoform (P49662) was detected at almost identical levels in both cell lines. It is part of an incomplete protein pair with a ∆ = 5-fold difference between the ratio values of the two peptide nodes. This discrepancy in ratio values predicts that an autocatalytic cleavage product (E9PMT1), which covers the N-terminal segment of full-length caspase-4, accumulated in CFBEo^−^ cells. Similarly, a full-length ephrin type B receptor (P29323) proteoform was increased 10-fold in CFBEo^−^ cells, while a shorter proteoform of the same receptor was only twofold more abundant and its presence was not validated by a unique peptide node in the experiment. An ∆ = 8-fold discrepancy in ratio values of the peptide nodes suggests that more full-length than endogenously cleaved ephrin type B receptor accumulated in CFBEo^−^ cells.

**Fig. 6:**
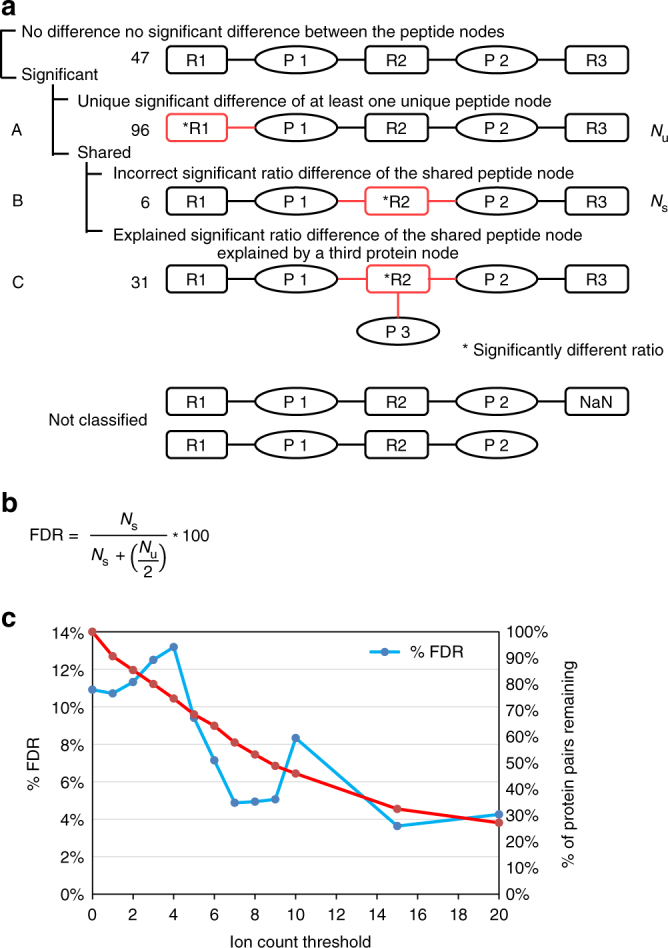
Classification of protein pairs based on user-defined settings and FDR calculation. **a** Protein pairs can be differentiated depending on whether unique or shared peptide nodes are significantly regulated, for example, are differentially expressed by more than a user-defined threshold. Significantly altered peptide ratios are outlined in red and connected to the corresponding protein node by an edge in red. Shared peptide nodes are not expected to be significantly regulated (incorrect) unless the shared peptide node connects to more than two protein nodes (explained). Protein pairs were not further considered in case a peptide node was missing or not determined (not classified). **b** An FDR is calculated according to the equation depicted. The number of protein pairs that include a shared peptide group that is significantly regulated (*N*_s_) is used to estimate the number of falsely discovered protein pairs that include at least one significantly regulated unique peptide group (*N*_u_). **c** The plot shows the relationship between number of protein pairs detected and corresponding FDR to ion count threshold settings. Abbreviations: P: protein, R: ratio

Furthermore, we analyzed all 180 complete protein pairs that were present in this proteomic dataset (Supplementary Data 7). We asked whether one of the two protein nodes would differ in relative abundance by a ∆ ≥ 2-fold threshold. Filtering with a threshold of ∆ ≥ 2-fold selected 133 of 180 protein pairs (74%, Supplementary Note 5). Because complete protein pairs were often embedded in more complex peptide-to-protein clusters, complete protein pairs were assigned to three different categories (Fig. 6a): Category “A” subsumed 96 complete protein pairs that had at least one unique peptide node measured with a discrepant ratio value. If the ratio value of the shared peptide node was discrepant relative to the ratio values of both unique peptides, the protein pair was placed either in category “B” (six protein pairs) or in category “C” if the shared peptide node was connected to a third protein node (31 protein pairs). A third protein node might explain the difference in ratio value of the shared peptide node in category “C,” whereas it remains unexplained in category “B.” Because shared peptide nodes in category “B” were most likely quantified incorrectly, a false discovery rate (FDR) for complete protein pairs with a significantly regulated unique peptide node can be deduced (Fig. 6b). Taking into account that the probability for unique peptide nodes to differ is twice that of the shared peptide node, we determined an FDR of 12.5%. Thus, at least 8 of 96 complete protein pairs with a ∆ ≥ 2-fold may be false positives.

The FDR value decreased with stricter filter settings (Supplementary Data 1). For example, requiring >7 isobaric counts per peptide node reduced the FDR more than twofold to 4.9%, while it reduced the number of complete protein pairs by 25% to 139 (72 unique) in 2747 connected components (Fig. 6, "Network 6”, Supplementary Data 8). Not unexpectedly, an increase in filtering stringency decreases the FDR; however, this inverse correlation is not linear, and, depending on the actual data, FDR values might show local minima with increasingly stricter filter settings.

### Analysis of individual protein clusters

**Fig. 7:**
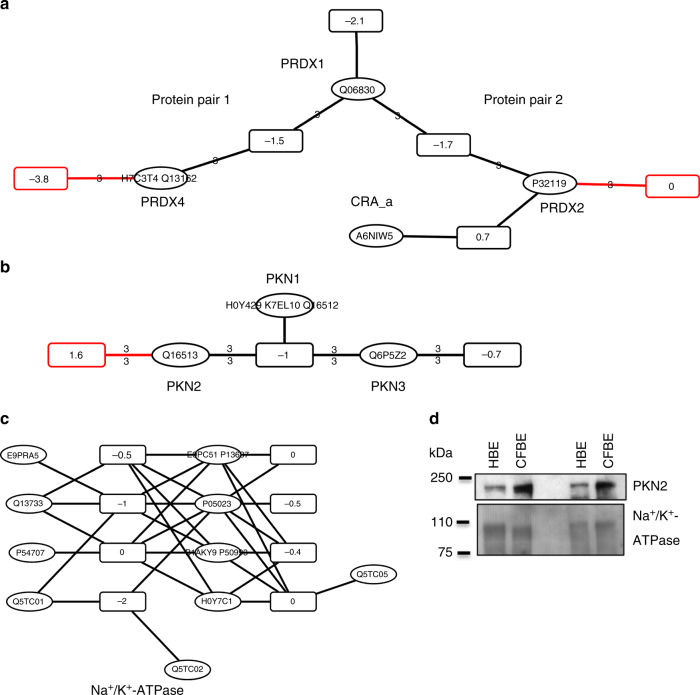
Select peptide-to-protein clusters with proteins differentially regulated in CFBE41o^–^ vs. HBE41o^–^ cells. **a** The protein cluster shows the family of related peroxiredoxin (PRDX) proteins. Edges and peptide node outlines in red as well as number associated with the edge in the peptide-to-protein clusters indicate differential expression according to the protein pair-centric analysis. **b** The protein cluster of serine–threonine kinase-related protein kinases PKN1, PKN2, and PKN3 is displayed. **c** The peptide-to-protein cluster of Na/K-ATPase proteins is depicted. It is expressed at the same levels in HBE41o^–^ and CFBE41o^–^ cells. **d** Western blot analysis was used to verify the difference in PKN2 expression levels between HBE41o^–^ and CFBE41o^–^ cells. Na^+^/K^+^-ATPase expression levels are shown as a loading control. Data in the western blot represent independent biological replicates, CFBE41o^–^: *n* = 2, HBE41o^–^: *n* = 2

The family of peroxiredoxin (PRDX) proteins exemplifies how a protein pair-centric analysis highlights differential expression of proteins within a protein cluster. There were two complete protein pairs within the cluster of PRDX-like proteins with a ∆ ≥ 2-fold. PRDX1 and PRDX4 expression decreased in CFBE41o^−^ cells over HBE41o^−^ cells, whereas PRDX2 was found in equal amounts (Fig. 7a). PRDX4 was more depleted relative to PRDX1 in one of the protein pairs. In the other protein pair, PRDX2 was up-regulated relative to PRDX1. The differences in expression between PRDX1 and PRDX2 as well as between PRDX1 and PRDX4 were classified as significant in protein pair analysis. In contrast, averaging over all peptide node ratios in the peptide-to-protein cluster would have indicated that all PRDX proteins were overall 2.5-fold down regulated but would have missed that PRDX2 was not down-regulated. Also, a maximum parsimony approach suggested down-regulation of PRDX1 only, whereas using unique peptide nodes only indicated no regulation for PRDX2 and down-regulation of PRDX1 and PRDX4. The cluster comprises one incomplete protein pair, PRDX2 and CRA_a, but because the difference in ratio values between the peptide node unique to PRDX2 and the shared peptide node was small (∆ < 2-fold), the relative abundance of CRA_a was only slightly different from PRDX2, in case CRA_a is present at all.

Finally, a serine/threonine-protein kinase (PKN) cluster comprising PKN1 (Q16512), PKN2 (Q16513), and PKN3 (Q6P5Z2) was identified. PKN2 (PRK2) shares 59% sequence identity with PKN1 and was three-fold more abundant in CFBE41o^−^ cells (Fig. 7b). Western blot confirmed that PKN2 was more abundant in CFBE41o^−^ cells when normalized to the expression levels of Na^+^/K^+^-ATPase (Fig. 7c and 7d, Supplementary Fig. 12). The regulation of PKN2 would have been missed with a quantification approach that takes all peptides into account, irrespective of whether or not they are unique to the proteoform. Alternative approaches in proteomic data analysis either failed to identify PKN2 as differentially regulated^30^ or underestimated the difference in expression^31^. A difference in PKN2 protein quantity may contribute to the altered cell morphology between HBE41o^−^ and CFBE41o^–^ cells in cell culture because bronchial epithelial airway cells require PKN2 to establish apical junctions^32^. PKN1 is part of two incomplete protein pairs with either PKN2 or PKN3, which provide conflicting evidence for a potential presence of PKN1. While the incomplete protein pair with PKN2 shows discrepant ratio values for the peptide nodes (∆ ≥ 2-fold), the incomplete protein pair with PKN3 shows similar ratio values (∆ < 2-fold).

## Discussion

We show that analysis of bipartite peptide-to-protein networks with PCQ can be used to uncover the presence of protein nodes and proteoforms. This approach is based on evidence that proteins and proteoforms in one set of a bipartite network can be quantified indirectly using measurement values of peptides in the second set. Bipartite peptide-to-protein networks have two advantages: first, each peptide is represented and quantified exactly once, and second, peptide nodes are tested for whether their relative abundance significantly differs from other peptide nodes within the same connected component. Thus, inferring the presence of proteoforms within a peptide-to-protein network increases proteome coverage and analysis.

We show that the presence of additional nodes in one set (protein nodes) can be correctly deduced from a difference in quantifications in the second set (peptide nodes). As proof of principle, in a two-species comparison we showed that 58% of incomplete protein pairs with discrepant ratio values were completed, whereas only 21% of incomplete protein pairs were completed when ratio values were congruent. Therefore, the presence of an additional protein node was deduced a from a discrepancy in ratio values with a four-fold increased likelihood. When comparing two isogenic cell lines, we found that caspase-4 and ephrin type B receptor protein fragments were differentially regulated relative to their longer proteoforms, respectively. These protein fragments are otherwise difficult to trace, and their regulation may reveal important biological insights. Thus, analyzing incomplete protein pairs can help to generate evidence for the presence of a protein node. However, a close inspection of the respective connected component might be required, and additional follow-up experiments are needed to prove the presence of a proteoform.

The sensitivity of this in-depth analysis depends heavily on the method of quantification and the quality of data, so a careful assessment of systematic and random errors in quantitation is recommended. PCQ analyzes MS2-based and MS1-based peptide quantification data, for example, upon stable isotope labeling by amino acids in cell culture^33^ taking measurement variability into account (Supplementary Note 6). By filtering out random errors due to chemical noise in MS2-based peptide quantification, we reduced the number of incorrectly quantified peptide nodes from 14 to 0.4%. Additional sources of error include imprecise *m*/*z* measurements, incomplete database entries, and spectra that are ambiguous in peptide identification (Supplementary Note 7). Notably, tri-partite network representations have been proposed to address the problem of ambiguous peptide identifications, for example, due to mass-neutral changes in the peptide sequence. Fragment ion spectra may be represented in a third set of nodes that may connect to multiple peptide identifications^34^. Thus, possible ambiguities in peptide to spectrum assignments also currently limit infering the actual presence of proteins in biological samples.

We implemented a novel FDR calculation for significantly regulated proteoforms within complete protein pairs. This FDR calculation relies on analyzing the connectivity within the connected component and thus dependents on database size. It is also limited in sensitivity by the complexity of the proteome identified. The accuracy of the FDR calculation will scale with database as well as proteome size and thus might be of limited use in conjunction with samples of low complexity.

In conclusion, bipartite network analysis with PCQ improves on existing methods for identifying and quantifying proteoform-specific differences between two proteomes. Additional analyses of peptide-to-protein networks can be easily performed, opening a new range of possibilities for comparing peptide-to-protein clusters and complete proteomes in complex multi-species settings^35^. The analysis of bipartite networks for significantly regulated nodes in one set of vertices based on quantitative values in the other set of vertices is of broad applicability. Indeed, it predicts the presence of nodes and pinpoints nodes that significantly deviate in relative weight in bipartite networks. PCQ might be of interest in fields of research other than proteomics that utilize bipartite networks.

## Methods

### *Drosophila* strains and sample preparation

*Drosophila* *melanogaster* (y[1]; Gr22b[1] Gr22d[1] cn[1] CG33964[R4.2] bw[1] sp[1]; LysC[1] MstProx[1] GstD5[1] Rh6[1]) and *D*. *virilis* (b[1]; tb[1] gp-L2[1]; cd[1]; pe[1]) fly strains were obtained from the UCSD Drosophila Stock Center and kept under standard husbandry^36^. Fly strains are inbred and thus isogenic to the respective reference fly strain used for whole genome sequencing and proteome assembly published in ref^37^. Flies laid eggs onto agar juice plates and embryos were collected between 0 to 6 h after egg laying. Embryos were de-chorionated, mechanically crushed in a small douncer, and lysed in lysis buffer (50 mM Tris-HCl, pH 7.5, 250 mM NaCl, 0.5% IGEPAL CA-630, 1 mM EDTA, 1× Complete EDTA-free protease inhibitor cocktail (Roche), 1× Phosphatase Inhibitor Mix (Roche)). Proteins were methanol/chloroform precipitated, solubilized, digested with endoprotease LysC, and chemically labeled with isobaric isotopologues as described in Bamberger et al.^27^ The dataset includes two biological replicates that were each measured in technical triplicate.

### Cell culture and western blot

CFBE41o^–^ and HBE cells were cultured in advanced minimum essential medium (A-MEM, Gibco, Carlsbad, CA, USA) supplemented with 2 mM l-glutamine and 1% penicillin-streptomycin at 37 °C, 5% CO_2_. Cells were harvested at confluency in TNI lysis buffer (250 mM NaCl, 50 mM Tris-HCl, 1% Igepal-CA-630, 1 mM EDTA, 1× EDTA-free Complete Protease Inhibitor (Roche))^16^. For detection of proteins by western blot, cell lysate in 1× Laemmli SDS-sample buffer was heated to 95 °C for 5 min and separated on 8 or 10% Novex SDS acrylamide gels (Invitrogen). PKN2 and NaK-ATPase were detected by incubation with monoclonal anti-PKN2 antibody (dilution 1:1.000, clone 3A7, Novus Biologicals #H00005586-M01) and anti-Na^+^/K^+^ATPase antibody H-300 (dilution 1:2000, Santa Cruz #sc28800), respectively, followed by incubation with goat anti-mouse (dilution 1:10,000, Jackson ImmunoResearch Laboratories #205-035-108) or goat anti-rabbit (dilution 1:10,000, Cell Signaling Technology #7074S) IgG antibodies coupled to horse radish peroxidase, respectively. ARPE-19 cells were labeled heavy with ^15^N^13^C-lysine and ^15^N^13^C-arginine for >10 cell doublings.

### Mass spectrometry

Species-specific, isobaric isotopologue-labeled peptide samples of *D*. *melanogaster* and *D*. *virilis* were mixed in a 1:1 ratio, subsequently loaded onto a MudPIT column^38^ and analyzed either on an Orbitrap Velos Pro or an Orbitrap Fusion Tribrid mass spectrometer (Thermo) with the following settings: one full scan mass spectrum (*m*/*z *400–2000) was acquired at a resolution of R 60,000. Subsequent fragment ion spectra of the ten most abundant peaks were recorded in a data-dependent fashion. Each precursor ion was fragmented first with collision-induced fragmentation (CID) at 35% normalized collisional energy and an ion count threshold of 1000 and then with higher collisional energy-induced dissociation (HCD). HCD spectra were acquired for precursor ions with an ion count of >5000 and 40% normalized collisional energy and recorded with a mass resolution of R 30,000 to resolve isobaric isotopologues in the lower *m*/*z-*range. Non-labeled and heavy-labeled ARPE-19 cell lysates were mixed in a ratio of 1:1, digested with trypsin, loaded on a MudPIT column, and analyzed with an Orbitrap velos mass spectrometer. Top 10 precursor ions of the MS1 survey scan were selected and subsequently fragmented by CID. Every condition (lacZ, wt, R345W) in the fibulin-3 experiment was measured in biological triplicate (*n* = 3).

### In silico digest of reference databases

The UniProt database (UniprotKB/TrEMBL release 2014_05) with 30,043 proteins (*D*. *virilis*) and 30,296 proteins (*D*. *melanogaster*) was digested in silico with the endoprotease LysC assuming no missed cleavages, no cleavage between K and P, and including all 6 to 60 amino acids long proteolytic cleavage products (peptides).

The Human UniprotKB/TrEMBL release 2013_09 was used by PCQ for the CFBE41o^–^ vs. HBE41o^–^ proteomic comparison and the UniprotKB/TrEMBL release 2016_05 for the fibulin-3 dataset. Both databases were digested in silico with the endoprotease trypsin allowing for two missed cleavages and no cleavage between K and P. Proteolytic cleavage products (peptides) with a length from 6 to 60 amino acids were included in the databases.

### Analysis of mass spectrometric data

Mass spectra were extracted in an ASCII-text file format (.ms1 and .ms2) from raw data files with RawConverter^39^. RawConverter corrected each precursor ion peak to its mono-isotopic peak when appropriate. All tandem mass spectrometry (MS/MS) spectra were compared with the search algorithm ProLuCID^26^ against theoretical mass spectra calculated from the in silico digested reference databases (see above) using a decoy strategy^40^. Precursor mass tolerance was set to 50 ppm and fragment ion tolerance to 500 ppm for CID spectra with carbamido-methylation of cysteine as static modification. The identified PSMs were filtered to an FDR of ≤0.14% at a PSM level with DTASelect2^3^. The FDR was calculated based on the number of PSMs that matched sequences in the reverse decoy database of *D*. *virilis* and *D*. *melanogaster*. MS/MS spectra of human sample origin were searched with ProLuCID against the human UniprotKB/TrEMBL database mentioned above and including a list of standard contaminant proteins.

A modified version of the software package Census (census_ms2) was used to extract the intensities of isobaric isotopologue fragment ion peaks that matched the theoretical b-ion and y-ion fragment masses calculated for the identified peptide to determine the relative abundance of peptides. Peptide sequences and isobaric isotopologue information was uploaded to Proteomics INTegrator PINT. The average (mean) of isobaric isotopologue ratios per MS/MS spectrum (Ri) was calculated and the samples further analyzed following the approach published by Navarro et al.^28^ Alternatively, a count ratio Rc was calculated based on all technical replicates (Supplementary Method). The sample size was six and the data analysis pipeline was tested for its ability to accurately detect a predefined effect size, which is the ability to correctly reflect the species specificity of peptides in the quantitative results. The investigators were not blinded to the group allocation during the experiment or data analysis.

### Peptide-to-protein network assembly

PCQ, a new software implemented in Java, was designed and implemented to run all the analyses described here. As a first step in the analysis, a new “network-walking” algorithm was created to generate peptide-to-protein clusters, in which all potential proteins inferred from the detected peptides are clustered based on peptide-to-protein mappings and optionally based on peptide similarity (Supplementary Information). The program iterates over the complete peptide and protein set, assigning each peptide and protein to a cluster in which the peptides and proteins are connected by at least one edge. Eventually, all identified proteins sharing one or more peptide sequences are added to the same cluster. To reduce complexity in the network, the algorithm collapses peptides that connect to identical proteins into one peptide node and subsumes proteins that connect to the identical set of peptides into a single protein node in the network.

In addition, a minimum threshold of detected isotopic peaks per peptide (or peptide group) was created. The threshold allows filtering peptides by the number of isobaric isotopologue peaks independently of the number of PSMs. Additional filter options are available (Supplementary Information).

### Quantification of peptide nodes

Quantitative Rc values are based on the occurrence of either heavy or light isobaric isotopologues peaks in MS2-based quantification approaches. Rc is the ratio of all light over heavy isobaric isotopologue peaks detected in all PSMs for each peptide. To calculate a ratio for a peptide group (Rc_pg_), each ion count was normalized by the number of PSMs per peptide to correct for the differential fragmentation pattern and detection efficiency of individual peptides with different amino acid sequences (Supplementary Fig. 1c). If orthologs were present in identical amounts in each species-specific sample, the ratio value for a species-indifferent peptide was 1:1 or log_2_(1/1) = 0.

Alternatively, isobaric isotopologue ratio (Ri) quantifications in MS2-based (Supplementary information) or MS1-based peptide quantifications (fibulin-3 dataset) were analyzed with SanXoT (v.1.17)^28^ wherein the weight of each measurement is inversely proportional to its variance and an FDR reflects the likelihood of ratios to be false. Initial ratios were calculated based on average isobaric isotopologue ratios Ri per PSM (MS2) or based on the area under the chromatographic peak per PSM (MS1). PSM-based ratios were averaged per peptide and subsequently per peptide node. Initial fitting weights for the weighted averages were based on signal intensity *I*^2^. Within each aggregation step, all measurements were analyzed for outliers with a FDR threshold of <0.01 and outliers were not further considered (SanXoTSieve v.0.05).

### Network analysis

The peptide-to-protein network was analyzed to identify pairs of protein nodes (e.g., protein pairs) that share at least one peptide node and harbor unique peptide nodes associated with each protein node. All complete protein pairs were then analyzed for the presence of outliers among the ratios of the peptide nodes according to either statistical testing (Hoaglin and Iglewicz statistical test) or a predefined fold-change cutoff. When isobaric isotopologue labeling is used, either Rc or Ri can be considered. Alternatively, ratio values can be derived from MS1-based quantifications strategies or provided in a file in tabular format. Subsequently, all protein pairs are categorized and PCQ creates a text file that lists all protein pairs and classifications.

PCQ exports the peptide-to-protein networks in the eXtensible Graph Markup and Modeling Language file format which can be visualized in Cytoscape^21^ or other programs.

### Code availability

The complete code for ProteinClusterQuant is available from GIT-hub, https://github.com/proteomicsyates/ProteinClusterQuant as well as tutorials and a Wiki page with more information related to the software.

### Data availability

Mass spectra are available under MassiVE MSV000081535 and ProteomeXchange PXD007746. Peptide sequences and isobaric isotopologue information is available in Proteomics INTegrator (Supplementary Note 8). Peptide-to-protein networks are available through NDEx (Supplementary Note 9). All data relevant to this study is also freely available from the authors.

## Electronic supplementary material

Description of Additional Supplementary Files

Supplementary Information

Supplementary Data 1

Supplementary Data 2

Supplementary Data 3

Supplementary Data 4

Supplementary Data 5

Supplementary Data 6

Supplementary Data 7

Supplementary Data 8

Supplementary Data 9

Supplementary Data 10

Supplementary Data 11

Supplementary Data 12
