# Supplementary material for: Deducing the presence of proteins and proteoforms in quantitative proteomics
Source: Nat Commun. 2018 Jun 13;9:2320. doi: 10.1038/s41467-018-04411-5 (PMC5998138; doi:10.1038/s41467-018-04411-5)
Supplement: Supplementary file 1 — Description of Additional Supplementary Files [file 41467_2018_4411_MOESM1_ESM.pdf]

Descriptions of Additional Supplementary Files

File Name: Supplementary Dataset 1: Detailed list of PCQ parameter settings
Description: PCQ Parameter settings that were used to generate peptide-to-protein networks 1 through 9. Settings that changed between different network analyses on the same proteomic dataset are highlighted in yellow.

File Name: Supplementary Dataset 2: Non-collapsed network of D. melanogaster embryos versus D. virilis
Description: The peptide-to-protein network compares the proteome of D. melanogaster embryos with D. virilis embryos and was assembled with all quantified sequence-unique peptides and all proteins, including subset proteins.

File Name: Supplementary Dataset 3: Condensed network of D. melanogaster embryos versus D. virilis
Description: The peptide-to-protein network compares the proteome of D. melanogaster embryos with D. virilis embryos and was assembled with all quantified peptides collapsed into peptide groups and displayed as individual peptide nodes in case they are shared by the same protein nodes. All proteins are collapsed into protein groups according to the peptide sequences that they share.

File Name: Supplementary Dataset 4: Filtered network of D. melanogaster embryos versus D. virilis
Description: The peptide-to-protein network compares the proteome of D. melanogaster embryos with D. virilis embryos and was assembled with all quantified peptides collapsed into peptide groups and proteins collapsed into protein groups. Individual peptide nodes were included only if ≥ 2 isobaric ion counts were present.

File Name: Supplementary Dataset 5: All peptide network of D. melanogaster embryos versus D. virilis
Description: The peptide-to-protein network compares the proteome of D. melanogaster embryos with D. virilis embryos and was assembled irrespective of whether peptides were quantified. Individual peptide nodes were included in quantitation only if ≥ 2 isobaric ion counts were present.

File Name: Supplementary Dataset 6: Network of CFBE41o- versus HBEo- cells
Description: The peptide-to-protein network for the comparison of CFBE41o- with HBE41o- cells was assembled with all peptides collapsed into peptide groups and proteins collapsed in protein groups. Individual peptide nodes were included only if ≥ 2 isobaric ion counts were present per node. The user-defined fold change threshold for the classification scheme was set to > 2-fold.

File Name: Supplementary Dataset 7: List of all complete protein pairs in CFBE41o– versus HBE41o–
Description: All complete protein pairs that were classified in the CFBE41o– versus HBE41o– protein-peptide network with an ion count threshold of >2 are listed. A user-defined significance threshold was set to > 2-fold.

File Name: Supplementary Dataset 8: Filtered network of CFBE41o- versus HBEo- cells
Description: The peptide-to-protein network for the comparison of CFBE41o- with HBE o- cells was assembled with all peptides collapsed into peptide groups and proteins collapsed in protein groups. Individual peptide nodes were included only if ≥ 7 isobaric ion counts were present per node. The user-defined fold change threshold for the classification scheme was set to > 2-fold.

File Name: Supplementary Dataset 9: Peptide network of D. melanogaster embryos versus D. virilis

Description: The peptide-to-protein network compares the proteome of D. melanogaster embryos with D. virilis embryos and was assembled with all proteins collapsed into protein groups according to the peptide sequences that they share. Peptide nodes with amino acid sequences of ≥ 80% similarity are connected by a green edge. (cited in Supplementary Methods).

File Name: Supplementary Dataset 10: Network of R345W Fibulin-3 overexpressing cells versus control
Description: The peptide-to-protein network compares R345W Fibulin-3 overexpressing cells with controls (nontransfected and SILAC labeled heavy) and was assembled with peptides collapsed into peptide groups and proteins collapsed into protein groups. Individual peptide nodes were included only if ≥ 2 spectral counts were present. (cited in Supplementary Methods).

File Name: Supplementary Dataset 11: Network of wt Fibulin-3 overexpressing cells versus control
Description: The peptide-to-protein network compares wt Fibulin-3 overexpressing cells with controls (non-transfected and SILAC labeled heavy) and was assembled with peptides collapsed into peptide groups and proteins collapsed into protein groups. Individual peptide nodes were included only if ≥ 2 spectral counts were present. (cited in Supplementary Methods).

File Name: Supplementary Dataset 12: Network of lacZ overexpressing cells versus control
Description: The peptide-to-protein network compares lacZ overexpressing cells with controls (non-transfected and SILAC labeled heavy) and was assembled with peptides collapsed into peptide groups and proteins collapsed into protein groups. Individual peptide nodes were included only if ≥ 2 spectral counts were present. (cited in Supplementary Methods).
