# Supplementary material for: Deducing the presence of proteins and proteoforms in quantitative proteomics
Source: Nat Commun. 2018 Jun 13;9:2320. doi: 10.1038/s41467-018-04411-5 (PMC5998138; doi:10.1038/s41467-018-04411-5)
Supplement: Supplementary file 2 — Supplementary Information [file 41467_2018_4411_MOESM2_ESM.docx]

# Supplementary Information to Deducing the Presence of Proteins and Proteoforms in Quantitative Proteomics

Bamberger *et al.*

# Supplementary Methods

## Quantitation of relative peptide abundance

Di-methylation of primary amines with isobaric isotopologues introduced a 5.64 mDa mass difference between light and heavy labeled peptides per primary amine (Supplementary Figure 1).  As previously shown ^1^, the small mass difference of the isobaric isotopologues was resolved in the low to mid *m/z*-range of MS/MS spectra of fragment ions recorded at a resolution of 30,000.  Thus, each peptide fragment ion peak is composed of light and heavy isobaric isotopologues. This experimental setup allows several ratio measurements per peptide spectrum match (PSM), thereby greatly enhancing quantification robustness.

## Determination of isobaric ratio (*Ri*)

A mean *isobaric ratio* (*Ri*) was calculated per each peptide for quantification (Supplementary Figure 1). The ratio was based on all of the isobaric isotopologue ion pairs identified in an MS/MS spectrum (Supplementary Figure 1a).  For each PSM, an average (mean) ratio was calculated from the individual ratios of the isobaric isotopologue fragment ion pairs.  In the same way, a ratio (mean) for each peptide was calculated from the ratio values obtained for each PSM.  The ratio aggregation through the different levels (ion pair to PSM to peptide to peptide node) included error propagation and statistical data analysis according to the statistical framework (SanXoT) suggested by Navarro *et al.* ^2^.  According to SanXoT the aggregation of the ratios from each level to the next is performed based on the weighted average of ratios in the previous level, wherein the weight is inversely proportional to the standard deviation.  For the analysis of the isobaric isotopologue labeling in MS/MS spectra we’ve applied a fitted weight at fragment ion level. It ensured that the null hypothesis of the normal distribution is fulfilled as best as possible.  A standard propagation of measurement errors enables that the relative abundance of individual peptides as well as peptide nodes is determined with high precision.  The error of measurement for each individual peptide node is listed in either the text-file output or the Cytoscape node, along with false discovery rate (FDR) value that is reported by SanXot.

## Determination of *count ratio* (*Rc*)

In cases where isobaric ion pairs and/or light only and/or heavy only isobaric isotopologues are present in the same spectrum for different fragment ions, an isobaric ratio *Ri* calculation cannot be accomplished (Supplementary Figure 1b). Additionally, for individual PSMs, peptides, or peptide nodes it is possible that different quantitative measurements need to be aggregated (e.g. ratio values with +∞ and/or –∞) which precludes consistent *Ri* calculations. Therefore, we introduced a *count ratio* (*Rc*) per peptide.  The count ratio *Rc* is based on the counts of light over heavy isobaric isotopologue peaks of all PSMs per peptide (Supplementary Figure 1b).  For a group of peptides within a single peptide node, an average count ratio per peptide node (*Rc*_pg_) was derived from the count ratio values of individual peptides (Supplementary Figure 1c).  The ion counts were normalized to the number of PSMs of each peptide in order to compensate for differences in chemical properties between peptides, which can further influence detection efficiency and fragmentation pattern.  All ratio values are reported as log_2_-transformed values.

In the species comparison experiment we often detected peptide fragment ions as a light or heavy isobaric isotopologue only, which precluded calculating a ratio value (Supplementary Figure 1d and 1e).  In these cases, PSMs were assigned positive or negative infinities (denoted as “INF”), corresponding to the log_2_-transformed ratio values of infinity (light only) or zero (heavy only), respectively.

## Peptide alignment and reporting peptide-peptide edges in PCQ

The degree of similarity between different peptide spectrum matches was assessed using the Needleman-Wunsch algorithm ^3^, and peptides were scored based on the likelihood of amino acid substitutions according to the BLOSUM62 matrix ^4^.  The score reported by the Needleman-Wunsch algorithm and the degree of identity between the peptides were used to find and group orthologous proteins together.  For the Drosophila dataset, pairwise alignments were filtered by the percentage of identity and alignment score (Supplementary Figure 2).  Peptides pairs with ≥6 amino acids consecutively aligned, an alignment score of ≥30 (<0.042% of all alignments), and a sequence similarity of ≥80% (<0.14% of all alignments) were retained, and this information included in the peptide-to-protein network as a peptide-peptide edge.  Filter settings were based on initial search of MS/MS data and require a minimum length of 6 amino acid sequence for the search, the alignment score to be set >2.5 sd from the mean and sequence similarity to be set to >5 sd from mean.

## K-means clustering

Alternatively, complete protein pairs can be clustered by k-means clustering to identify co-regulated subsets of proteins pairs that show similar regulation.  All peptide node measurements per protein pair were analyzed by MATLAB’s k-means clustering ^5^.  Protein pairs with a variance of more than the 10^th^ percentile were retained for k-means clustering which identified 67 (32%) protein pairs with a threshold ≥3 ion counts, and 37 (28%) protein pairs with a threshold ≥8 ion counts as differentially regulated.  Protein pairs were grouped in 9 different sub-clusters representing distinct profiles (Supplementary Figure 3a and 3b).  Protein pairs can also be analyzed for the presence of one of the protein nodes in only one of the two samples.  Indeed, in 8 protein pairs one protein node was present solely in either CFBE41o^-^ or HBE41o^-^ cells (Supplementary Figure 3c).

# Supplementary Notes

## Supplementary Note 1

### Dispersion of ratio measurements that deviate from 1:1

To test PCQ, a whole cell proteome isolated from a HEK cell lysate was digested, peptides labeled light as well as heavy with dimethyl labeling, mixed 1:1 and analyzed with mass spectrometry as previously described ^1^. Data analysis with PCQ based on isobaric ratios (*Ri*) revealed the dispersion of relative peptide abundances that are not matching the expected ratio of 1:1 (Supplementary Figure 4). When comparing technical replicates (HEK cell lysate), the standard deviation of all peptide ratio measurements was 0.7 (log_2_-fold change). When comparing the proteomes of two different drosophila species, isobaric ratios (*Ri*) were more spread out with a standard deviation of 2.0 (log_2_-fold change). As expected, the difference in standard deviation indicates that differences in protein expression between the two Drosophila species is higher than the variation found between technical replicate measurements of the same proteome (here: HEK proteome).

### Estimation of chemical background noise as a source of error

Background noise in the fragment ion spectrum can skew fragment ion peak quantification results and lead to erroneous ratio values.  In order to determine the number of incorrect quantifications, the proteome of one D. *virilis* embryo was labeled heavy and analyzed on a ThermoFisher VelosPro Orbitrap mass spectrometer (Supplementary Table 1).  475 different peptides were quantified and 2.8% were detected with light isotopologues in one or several spectra.  Thus, erroneous quantification of the isobaric isotopologues due to chemical noise for this experiment is ~3%.

## Supplementary Note 2

### Predicting missing proteins in the Drosophila species comparison

38 incomplete protein pairs were detected in the Drosophila species comparison.  24 incomplete protein pairs had infinity and ratio measurements as expected, according to species specificity and proteins identified.  Of these, 58% converted into complete protein pairs when adding identified but not quantified peptides to the peptide-to-protein network.  Additional 14 incomplete protein pairs were identified with congruent ratio or infinity values (Supplementary Figure 5).  3 incomplete protein pairs included +∞ or –∞ measurements for the shared peptide node, which is a possible but unlikely outcome of the experiment.  Additional, 3 incomplete protein pairs included unique peptide nodes with large ratio values which is most likely a designated infinity value that turned into a ratio measurement due to the presence of chemical noise (see above).  6 protein pairs were not expected to be completed:  5 included protein nodes that subsumed orthologues of both species and 1 comprised proteins present in one species only.  The ratio values of 2 additional protein pairs remained unexplained.

3 of these 14 congruent but incomplete, protein pairs were completed by non-quantified peptides: 2 had congruent infinity measurements (green boxes in Supplementary Figure 5) and the final 1 included a unique peptide node with an extreme ratio value which is most likely as a result of chemical noise (Supplementary Figure 5, circled in green).

## Supplementary Note 3

### LMO7 peptide-to-protein cluster in HBEo^-^ cells

LIM domain only protein 7 (LMO7) proteoforms were found in HBE41o^–^ cells and not in CFBE41o^–^ cells (Supplementary Figure 6), confirming previous observations ^6^.  LMO7 proteoforms contain several PDZ domains, including a Calponin homology domain and a LIM domain, and are thought to be involved in ubiquitination and proteasomal degradation of proteins for antigen processing.  LMO7 is preferentially expressed in bronchial epithelial airway cells where it localizes to *zona adherens* on the apical side of differentiated airway cells ^7^.  Mice lacking LMO7 slowly develop spontaneous adenocarcinomas at the termini of respiratory bronchioles ^8^.  LMO7 was also described as a *bona fide* interactor of CFTR ^9^.  Neither the structure of the LMO7 cluster nor the quantitative values in each peptide node (–INF) provide conclusive information whether one or several LMO7 proteoforms are present in the sample.  A maximum parsimony approach argues for the protein node with most peptide-to-protein edges (three) to be the most likely proteoforms present in the sample (F8WD26, J3KP06, Q8WWI1).  Here, PCQ reports one average abundance ratio for the peptide-to-protein cluster.

## Supplementary Note 4

### Predicting missing proteoforms in the HBEo- to CFBEo- comparison

712 connected components in the bipartite network were incomplete protein pairs which were devoid of one unique peptide node.  To find incomplete protein pairs with discrepant ratio values, the difference was required to be more than 4.85-fold which is two standard deviations of the overall average.  26 incomplete protein pairs passed this filter criterion.

## Supplementary Note 5

### Identification of differentially regulated protein pairs

A fixed threshold (minimal fold change) value was implemented to allow users to identify differentially regulated proteins within either a peptide-to-protein cluster or a protein pair.  One advantage of the user-defined cutoff is that infinity values are considered along with ratio values. This is not available in statistical testing for inequality.  Protein pairs are analyzed for the two unique peptide values and the shared peptide values. When setting a user-defined threshold, all protein pairs are analyzed for having at least one peptide node that differs by more than a fixed fold-change from the other two peptide nodes.

## Supplementary Note 6

### Differences in protein expression measured with stable isotope labeling *in vivo*

PCQ reports a FDR estimation for all peptide nodes in the bipartite network based on the method implemented in SanXoT.  SanXoT takes the type of isotope labeling and subsequent quantification based on MS survey scan (MS1) or MS fragment ion scan into account when calculating the FDR for peptide nodes. This is exemplified with a dataset based on a stable isotope labeled amino acids in cell culture (SILAC).  Here, acquisition and measurement of relative peptide abundances differed from the two previous examples: Chromatographic peak areas of peptides were determined based on successive MS1-scan ion intensities and the ratio between light and heavy areas of the isotope labeled peptide was calculated.  The experiment was designed to find changes in protein expression in the proteome of ARPE-19 cells upon overexpression of mutated fibulin-3 (EFEMP1).  Fibulin-3 is a secreted glycoprotein of the fibulin family of extracellular proteins.  An R345W mutation in fibulin-3 causes excessive extracellular deposits of fibulin-3 as well as other proteins and lipids between Bruch’s membrane and the retinal pigment epithelium that variably results in an inherited macular degeneration called Doyne honeycomb retinal dystrophy/Malattia Leventinese.  R345W fibulin-3 knockin mice ^10^ fully recapitulate this characteristic of the human disease and while loss of fibulin-3 function is not causative to the disease ^11^, genetic linkage studies suggested that activation of complement cascade and inflammation are key events in the molecular pathology of the disease ^12^.  Overexpression of R345W fibulin-3 in ARPE-19 cells leads to increased retention of the mutated protein at the endoplasmatic reticulum and intracellular accumulation ^10,13,14^.  R345W mutated fibulin-3 binds to calnexin, GRP78 and GRP94 ^15^.

The proteome of ARPE-19 cells overexpressing R345W ([Network 8](http://www.ndexbio.org/#/network/255e28af-ce1d-11e6-86b1-0ac135e8bacf?accesskey=84b904e6dedf21ad4a77783965ffbff42a6ac8f845dc469e7aae5aedfd7d9b30), Supplementary Data 10) or wt fibulin-3 ([Network 9](http://www.ndexbio.org/#/network/65fcc7fe-ce1c-11e6-86b1-0ac135e8bacf?accesskey=d9f33774f4a6c3b21b6ab391676184690f85bb84f96b175fe3c5b8015f62822e), Supplementary Data 11) was analyzed and compared to ARPE-19 cells overexpressing lacZ as control ([Network 10](http://www.ndexbio.org/#/network/2f34316d-ce1a-11e6-86b1-0ac135e8bacf?accesskey=aa5d86df222e286770f2cc4cff32168de1f1c3005d06dddf20297d290bafd89d), Supplementary Data 12).  Proteins in each experimental condition were labeled light with isotope-defined amino acids and quantified relative to uninfected ARPE-19 cells that were labeled heavy (Supplementary Figure 7a).  Census was used to determine peptide ratios ^16^ and proteomes were assembled in peptide-to-protein networks with PCQ which were filtered for >2 ratio measurements per peptide node.

Endogenous fibulin-3 was constitutively expressed in ARPE-19 cells.  2.8-fold more fibulin-3 was observed in wt fibulin-3 overexpressing cells.  Mutated fibulin-3 is known to accumulate and R345W fibulin-3 was 6.5-fold more abundant in ARPE-19 cells when compared to endogenous fibulin-3 levels.  The 2-fold upregulation of mutated over wt overexpressed Filbulin-3 served as a positive control.  Additional proteins that changed only upon over-expression of mutated Fibulin-3 but not wt or lacZ were identified by comparing significantly regulated genes (FDR <0.05) between all three conditions (Supplementary Figure 7b).  10 gene clusters unique to mutated Fibulin-3 over-expressing cells were detected with a >4-fold change in at least one peptide node harboring at least two different peptides (Supplementary Figure 7c).  Notably, overexpression of R345W Fibulin-3 in ARPE-19 cells caused up-regulation in a C-terminal proteoform of Ubiquitin ligase UBE4A (B7Z7P0) which mediates proteasomal degradation of proteins.

## Supplementary Note 7

### Assessment of quantification accuracy

To assess the accuracy of isobaric isotopologue ratio measurements in more detail, the proteome of one or several embryos were digested and labeled light (D. *melanogaster*) or heavy (D. *virilis*), respectively.  After labeling, both species-specific samples were mixed together and a sample quantity corresponding to one embryo was analyzed.  In addition, a “label-swap” experiment was performed, in which D. *virilis* was labeled light and D. *melanogaster* heavy.  All samples were measured in technical triplicates on a ThermoFisher Orbitrap Fusion mass spectrometer.

The proteomes identified in each technical replicate overlapped by >70% with the two additional replicates (Supplementary Figure 8).  Isobaric ratio *Ri* measurements were reproducible in the label swap experiment, with a coefficient of determination (R^2^) of 0.72 (Supplementary Figure 9a).  Further, we determined whether count ratios (*Rc*) matched the corresponding isobaric ratios (*Ri*) for each peptide (Supplementary Figure 9b).  An R^2^ of 0.82 indicated an existent but limited correlation between isobaric and count ratio measurements.  Indeed, the precision of the count ratio measurement heavily depends upon the sum of all counts per ratio calculation:  when only a small number of isobaric isotopologues is detected, ratio calculations close to 1:1 are less precise, whereas isobaric ratio calculations derived from a larger number of isobaric isotopologues are expected to be more precise.  Thus, the use of isobaric ratios versus count ratios depends on the specific experimental parameters.

Further, we analyzed the dataset for isobaric ratios that did not match the predicted species-specificity in order to evaluate the precision of ratio measurement.  In total 391 peptides were quantified with an isobaric ratio *Ri*.  13% (52 peptides) of these were species-specific, as judged from their amino acid sequence (Supplementary Figure 9c).  A subset of 8 species-specific peptides were deemed significantly regulated (FDR <5%).  All 8 peptides had extreme ratio measurements (>20-fold difference), indicating that the peptides are indeed species-specific.  Moreover, the significant *Ri* values matched the species specificity: one D. *virilis*-specific peptide was found to be negative (red circle) and 7 D. *melanogaster* peptides were positive (red squares).  Therefore, our data indicates that relative ratio measurements of >20-fold are of low reliability because a peptide measured with a ratio of >20-fold might be in fact present in only one of the two samples.  Chemical noise during acquisition of mass spectra generates isobaric isotopologue ions with small signal intensities that can lead to the measurement of an extreme isobaric ratio for a species-specific peptide.

The additional 44 of 52 species-specific peptides were quantified with *Ri* values that were not deemed significantly regulated.  A subset of these peptides was measured with a fitting weight of <0.66. This cutoff value for a minimal fitting weight was set to exclude 10% of all peptides that were measured with very low precision, e.g. with very low ion signal intensity.  The remaining peptides were measured with a ratio close to 1:1 indicating that the peptide sequence is unexpectedly present in the other species.  Explanations for this observation might include mass neutral amino acid exchanges between two peptides, a missing database entry, a falsely identified peptide sequence, or post-translational modifications that alter the mass of a peptide to the mass of peptide with a very similar amino acid sequence.

We also analyzed the count ratios *Rc* that did not match the predicted species/specificity, as we did with the isobaric ratios. For this purpose, we observed the distributions of the count ratios and looked at the proportion of incorrect ratios, based on species specificity. Supplementary Figure 9d shows that incorrect measurements are mainly distributed over the extreme ratio values, with incorrect D. *melanogaster*-specific peptides (orange) are located towards the positive end of the distribution and D. *virilis*-specific peptides (red) are located towards the negative end of the distribution.

In summary, species-specificity and quantitation of peptides based on *Ri* ratio values matched in >90% of peptides.  Technical limitations like ion interference during mass spectrometric acquisition of data or isotope purity of the labeling reagent as well as missing database entries or chemical modification of peptides explained several of the erroneous quantitation results.

### Comparison of *in silico*, detected, and quantified peptidome

An *in silico* digest of the D. *melanogaster* (30,296 proteins) and D. *virilis* (30,043 proteins) proteome (UniProt version 2014_05) with the enzyme LysC showed that 9.7% of peptides are indistinguishable, whereas the remaining 90.3% of peptides are species-specific (Supplementary Figure 10a).  In the experiment performed, 24% of all peptide PSMs were detected as labeled both light and heavy (Supplementary Figure 10b).  This relative increase might reflect that when peptides are present in both species their precursor ion intensity is doubled, thus increasing their likelihood of detection and identification.  During quantification, the percentage of peptides containing both light and heavy labels was reduced to 16%, but this is still higher than the expected value from the *in silico* digest of 9.7% (Supplementary Figure 10c).  The lower percentage of peptides that could be quantified containing both isobars might reflect differences in orthologue expression between the two species or might be due to insufficient signal intensity or mass resolution to detect both heavy and light isobaric isotopologues as pairs simultaneously.

48% and 36% of all quantified PSMs were light only or heavy only, respectively (Supplementary Figure 10c).  While the percentage of light only quantifications matches well the expected quantification result from the *in silico* digest, the percentage of peptides detected and unique to D. *virilis* (heavy only) was 10.8% lower than expected.  Given that the identical (isogenic) fly strains were used to generate the reference genome and thus proteome database, it might indicate that an overall lower sequence coverage of the D. *virilis* genome resulted in a less well assembled proteome database.

### Errors due to a missing database entry

The proteome of D. *melanogaster* embryos was directly compared to the proteome of D. *virilis* in a set of three experiments.  Samples per experiment comprised 5 or 10 embryos of D. *melanogaster* labeled light and 5 or 10 embryos of D. *virilis* labeled heavy, respectively (Supplementary Table 2).  Each sample was analyzed on a ThermoFisher VelosPro Orbitrap mass spectrometer and was evaluated for the percentage of correctly quantified peptides. 94.8% the quantitation result matched the expected species specificity (e.g. the pre-specified effect size).

5.2% of all peptides were quantified as present in one species even though the amino acid sequence was present only in the species-specific database of the other species. This could be due to either a missing database entry in the respective other species or imprecise m/z measurements. The argument that the result is due to a missing database entry is supported by the fact that the rate for it occurring was slightly higher for D. *virilis* (~2.9%) than for D. *melanogaster* (~2.3%), consistent the observation that the D. *virilis* protein sequence database might not be as complete as for the more extensively studied model organism D. *melanogaster*.  For example, the peptide with the amino acid sequence TLLNTSPNLLPITTATDTFNNLK is present only in the D. *melanogaster* database, e.g. peptides corresponding to this sequence should theoretically only be labeled light.  However, this peptide sequence was quantified as present in the D. *virilis* protein sample in about equal amount as in the D. *melanogaster* proteome (Supplementary Table 3).

### Random measurement errors

Alternatively, imprecise m/z value measurements can cause a systematic mass deviation of most or all fragment ion peaks in the spectrum from light to heavy or *vice versa*. A fraction or a complete spectrum, that deviates systematically in its m/z values, can lead to incorrect quantification and thus a mismatch between the ratio measurement and species specificity. Since a systematic mass deviation of a spectrum can occur randomly in either direction, peptide peak quantifications fail to match species specificity in almost equal amounts for both species.

Also, we identified that a rare co-elution of two similar peptides with mass-neutral amino acid exchanges during chromatographic separation may contribute to erroneous identification and quantification.  In one case, we found that chemical modifications on two species specific peptides resulted in peptides with almost identical precursor ion mass and very high amino acid sequence overlap (see example in Supplementary Table 4).

## Supplementary Note 7

### Including peptide similarity in the bipartite network

An optional analysis added peptide-peptide edges (light green) that connect peptides with a sequence similarity of ≥ 80% ((iii) in Figure 3b).  Overall, it included 183 new peptide-peptide edges in the network mainly within existing peptide-to-protein clusters, combined 50 orthologues and 10 paralogues otherwise present in distinct peptide-to-protein clusters, and thereby reduced the number of connected components to 343 (Supplementary Figure 11, [Network 6](http://www.ndexbio.org/#/network/1015fbb6-ce15-11e6-86b1-0ac135e8bacf?accesskey=6d49739ea0b50722ccf5085d80d6d3d568d746ef8fea4e931d954d005361a43b) and Supplementary Data 9).  One pair of orthologues (RpL35, Q9W499 and B4M807) remained separate because two peptides with high sequence similarity did not align for a minimal 6 consecutive amino acids.

Supplementary Note 8

Peptide sequences and isobaric isotopologue information is available in Proteomics INTegrator PINT for the ‘DmDv dataset:

[<http://sealion.scripps.edu/pint/?project=838f13f3da1bc24836e769d7c0eb9b69>]’, ‘CFBEvsHBE dataset [<http://sealion.scripps.edu/pint/?project=7cfe0b24282e72ce89562704ec63b4f1>]’ and ‘Fibulin dataset [<http://sealion.scripps.edu/pint/?project=e7b9ba8404b7d45810c182594b862b41>]’.

Supplementary Note 9

All subsequently listed peptide-to-protein networks are available through

NDEx [http://www.ndexbio.org)]:

**Network 1** [http://www.ndexbio.org/#/network/9240fd15-ce10-11e6-86b1-0ac135e8bacf?accesskey=aa92edeec7af2316bdd334498cb327228c99e2271a6874350023ec2cfa2bf463]

**Network 2** [http://www.ndexbio.org/#/network/22a9b0a7-ce15-11e6-86b1-0ac135e8bacf?accesskey=e6c21833cebfb0617c5fc6fdf9ce8b006b2e44c1675fb0f5788852ae64683d4d]

**Network 3** [http://www.ndexbio.org/#/network/33de4ac8-ce15-11e6-86b1-0ac135e8bacf?accesskey=85fe6b47eee96013aab20d8d72d2ada1ce1a8ad5388ac60aec5286acad117cfe]

**Network 4** [http://www.ndexbio.org/#/network/4a8f7739-ce15-11e6-86b1-0ac135e8bacf?accesskey=6d4fd258449afcce7f00f74304bf8b2d6a69ebfcdeac398fbf2e24c9f5c302fc]

**Network 5** [http://www.ndexbio.org/#/network/c637a79b-ce15-11e6-86b1-0ac135e8bacf?accesskey=52d97f23da00ec5885e193e218b14b033a0b2530be6b2f4ddf66b5d4e2055100]

**Network 6** [http://www.ndexbio.org/#/network/04e99cac-ce16-11e6-86b1-0ac135e8bacf?accesskey=16a82ef06e0b830b1db97f22c2b41ad83b3a123fc1ea317153952947d6d36997]

**Network 7** [http://www.ndexbio.org/#/network/1015fbb6-ce15-11e6-86b1-0ac135e8bacf?accesskey=6d49739ea0b50722ccf5085d80d6d3d568d746ef8fea4e931d954d005361a43b] (cited in Supplementary Methods)

**Network 8** [http://www.ndexbio.org/#/network/255e28af-ce1d-11e6-86b1-0ac135e8bacf?accesskey=84b904e6dedf21ad4a77783965ffbff42a6ac8f845dc469e7aae5aedfd7d9b30] (cited in Supplementary Methods)

**Network 9** [http://www.ndexbio.org/#/network/65fcc7fe-ce1c-11e6-86b1-0ac135e8bacf?accesskey=d9f33774f4a6c3b21b6ab391676184690f85bb84f96b175fe3c5b8015f62822e] (cited in Supplementary Methods)

**Network 10** [http://www.ndexbio.org/#/network/2f34316d-ce1a-11e6-86b1-0ac135e8bacf?accesskey=aa5d86df222e286770f2cc4cff32168de1f1c3005d06dddf20297d290bafd89d] (cited in Supplementary Methods).

# Supplementary Figures

**Supplementary Figure 1** **Quantitation of peptide groups labeled with isobaric isotopologues.  (a)** High resolution MS/MS spectra reveal the relative abundance of a peptide in each sample based on the presence and relative abundance of isobaric isotopologue fragment ion peaks.  When quantifying peptides, *isobaric ratios* were calculated from quantified isobaric isotopologue ratios normalized per PSM (*Ri*).  **(b)** Alternatively, a *count ratio* was calculated based on counting the presence of individual isobaric peaks that were either light or heavy in each peptide spectrum match (*Rc*).  **(c)** In cases where several peptides were subsumed into an identical peptide node, a count ratio per peptide group (*Rc*_pg_) was determined.  *Rc*_pg_ normalizes isobaric counts of individual peptides to the number of PSMs per peptide to take differential identification efficiencies into account.  **(d and e)**In case peptides are labeled light (green) only or heavy (red) only quantifications are log_2_(n/0) = +∞ or log_2_(0/n) = –∞, respectively.

**Supplementary Figure 2  Peptide sequence alignment and assembly of protein clusters based on peptide similarity.** The Needleman-Wunsch algorithm was used to align all combinations of the 1308 different peptides in the species-comparison dataset. The resulting individual alignment scores are plotted against the percentage of sequence identity obtained in each alignment.  Only peptide sequence alignments with high identity (≥0.8) or high sequence alignment score (≥30) were considered similar (183 peptide sequence alignments).

**Supplementary Figure 3  Identification of co-regulated protein pairs in the CFBE41o^–^ to HBE41o^–^ comparison.** Clustering of protein pairs based on k-means for protein pairs present in the dataset when filtered for >2 **(a)** or >7 **(b)** ion counts per peptide node**.** **(c)** The graphed values for the 8 protein pairs are shown.  Each line connects the first unique peptide ratio measurement with the shared peptide ratio measurement and finally with the second unique ratio measurement within one protein pair along the x-axis.  The actual ratio measurements are plotted along the y-axis.  Positive and negative infinity were set to the absolute maximum value in the dataset, 9.5 or -9.5, respectively.

**Supplementary Figure 4  Frequency distributions of *Ri* for a technical and a biological sample dataset.** The relative percentage of peptide nodes per fold change (*Ri*) is indicated (bin size 0.1) for the comparison of D. *melanogaster* with *D. virilis* dataset (green) as well as a comparison of an identical HEK cell lysate labeled heavy and light and mixed in a 1:1 ratio. A smoothed curve (width 5 data points) for each dataset is shown.****

**Supplementary Figure 5  Incomplete protein pairs with congruent peptide node values.** Connected components that represent incomplete protein pairs (consisting of 2 protein nodes and 2 peptide nodes) are displayed. 3 incomplete protein pairs were completed by including a unique peptide node that was not quantified (green circle and green box). The connected components are grouped according to their different characteristics. “Congruent infinity”, “species-indifferent protein node”, and protein pairs with a “single species” are plausible. A “large ratio value on unique peptide nodes” most likely is a consequence of chemical noise masking an infinity ratio. A small set of incomplete protein pairs remains “unexplained”.  Protein pairs of the D. *melanogaster* (blue ellipses), D. *virilis* (green ellipses), or species-indifferent (pink ellipses) protein nodes are shown.  The relative peptide abundance (*Rc*) is indicated in each peptide node wherein either a value indicates a relative log_2_(*Rc*) abundance in both species, +Infinity (red rectangles) its presence in only D. *melanogaster*, –Infinity (green rectangles) its presence in only *D. virilis*, and “N/A” that the peptide node was solely identified but not quantified in its relative abundance.

**Supplementary Figure 6  Protein cluster of LMO7 proteoforms which were found exclusively expressed in HBE41o^–^ cells.** Protein nodes are depicted as ellipses and their respective uniprotID is shown.  All peptide node measurements are –∞ (blue) and significantly different within the datatset (“*”).

**Supplementary Figure 7  Identification of R345W fibulin-3 co-regulated proteins. (a)** The schematic shows the experiment that was used to quire ARPE19 cells for R345W fibulin-3 co-regulated proteins. **(b)** Overlap of significantly regulated genes in either condition. Percentages are relative to all significantly regulated genes listed. **(c)** All peptide-to-protein clusters are shown that include significantly regulated peptide nodes with >1 peptide and of at least 4-fold difference and with an FDR of <0.01 and that are regulated in R345W only but not in wt fibulin-3 or lacZ overexpressing ARPE-19 cells.  Protein names of significantly regulated proteins are indicated.  A color scale indicates the relative abundance of the peptide nodes and a “*” denotes that the measurement was significant.  Abbreviations are: SILAC, stable isotope labeling with amino acids in cell culture; RP, reversed phase; SCX strong cation exchange; ESI, electrospray ionization.

**Supplementary Figure 8  Overlap in proteins between three technical replicates** of **(a)** D. *melanogaster* labeled light and D. *virilis* labeled heavy (“DmDv”) and **(b)** D. *virilis* labeled light and D. *melanogaster* labeled heavy (“DvDm”)*.*

**Supplementary Figure 9  Quantification of peptides based on isobaric ratios and reproducibility of measurements. (a)** The reproducibility of isobaric ratio measurements was assessed in a label swap experiment (n = 3 per condition).  The scatter plot shows the average isobaric ratio measured for each peptide if D. *melanogaster* was labeled light and D. *virilis* labeled heavy (“Dm/Dv”, y-axis) *versus* D. *virilis* labeled light and D. *melanogaster* labeled heavy (“Dv/Dm”, x-axis).  The least squared linear regression and coefficient of determination R^2^ are indicated.  **(b)** The correlation between isobaric ratio *Ri* and count ratio *Rc* for each peptide is shown with least squared linear regression line and coefficient of determination R^2^. **(c)**  The scatter plot shows isobaric ratios plotted against fitting weight for all peptides.  The fitting weight is the inverse of the corrected error of measurement.  Significantly regulated peptides with <5% false discovery rate (FDR) are indicated.  Abbr.: sign., significant.  n = 6.
**(d)** Histogram showing the frequency distribution of the ion count ratios (*Rc*) and the proportion of those peptides that were species-specific (red for D. *virilis*-specific peptides and orange for D. *melanogaster*-specific peptides). Because species-specific peptides are expected to be present in only one of the two samples, the ratio measurements indicated in red and orange can be considered incorrect.

**Supplementary Figure 10  Comparison of D. *melanogaster* with D. *virilis* proteomes by *in silico* digest, upon experimental identification, and quantification.**The Venn diagrams show the overlap of identical peptides and species-specific peptides normalized to the total number of peptides.  Proportion of peptides **(a)** *in silico* after digest with LysC, **(b)** as identified in the experiment, and **(c)** based on isobaric isotopologue quantification.  The percentage of each proportion of the Venn diagram is indicated as well as the species the proteome is derived from.  Averages are calculated from 3 technical replicates (b,c).  The average number of peptides (and standard deviation) detected is shown in brackets.

**Supplementary Figure 11** **All protein clusters are shown in which peptide similarity connects two orthologues that were separated in two independent connected components otherwise.** A peptide-peptide edge to indicate sequence similarity is shown in green.

**Supplementary Figure 12  Full-sized exposures of Western Blots shown in Figure 7.**

# Supplementary Tables

**Supplementary Table 1:  Absolute number of peptides detected by species-specificity in a single D. *virilis* embryo searched against both species-specific databases.**  Number of peptides detected in each experiment are shown.  Either a ratio measurement (*Rc*) or the result of the infinity measurement is shown, e.g. D. *melanogaster* specific peptides having a positive infinity value are indicated (D. *melanogaster*, +∞) as well as D. *virilis* peptides with negative infinity value (D. *virilis*, –∞).  The number of peptides with a quantification measurement signal but with a missing database entry indicated. **(a)** Experimental result of a single D. *virilis* embryo searched against both species-specific databases.

**Supplementary Table 2:  Results from comparing 5 (experiment A) or 10 (experiment B) embryos of each species.**  The absolute number of peptides detected by species-specificity and MS-based quantification are reported in the table.  Peptides that are present in D. *melanogaster* based on the quantitation result but that are not identified by a corresponding database entry in the D. *melanogaster* protein database are shown as “D. *mel* ID absent” or conversely as “D. *vir* ID absent”.

**Supplementary Table 3:  Individual peptide measurements with either incorrect isobaric isotopologue detection or with a missing database entry for the respective species.**  A low number of isobaric counts typically indicates an error in isobaric peak assignment to light or heavy, respectively, whereas a high number of unexpected isobaric counts such as in the first peptide indicates a missing database entry (peptide printed in bold).

**Supplementary Table 4: One amino acid (Q->E, indicated in bold red) exchange distinguishes the peptide sequence K.AQAAIAVEVAEALVK.A in the D. *melanogaster* orthologue of the ATP synthase, delta subunit, isoform A (Q9W2X6) from its D. *virilis* orthologue with the peptide sequence K.AEAAIAVEVAEALVK.A (B4M1Y1).**  Most likely, an additional post-translational modification resulted in incorrect sequence identification and quantitation for the D. *virilis* peptide sequence, as revealed by the counts of the light and heavy isobars in three out of 10 PSMs.  The non-modified *D. virilis* peptide was also identified with a mass difference of +1 Da (not shown).  The result might be explained by a posttranslational conversion of glutamate to glutamine.

# Supplementary References

1 Bamberger, C., Pankow, S., Park, S. K. & Yates, J. R., 3rd. Interference-free proteome quantification with MS/MS-based isobaric isotopologue detection. *Journal of proteome research* **13**, 1494-1501, doi:10.1021/pr401035z (2014).

2 Navarro, P. *et al.* General statistical framework for quantitative proteomics by stable isotope labeling. *Journal of proteome research* **13**, 1234-1247, doi:10.1021/pr4006958 (2014).

3 Needleman, S. B. & Wunsch, C. D. A general method applicable to the search for similarities in the amino acid sequence of two proteins. *J Mol Biol* **48**, 443-453 (1970).

4 Henikoff, S. & Henikoff, J. G. Amino acid substitution matrices from protein blocks. *Proceedings of the National Academy of Sciences of the United States of America* **89**, 10915-10919 (1992).

5 MacQueen, J. in *Proceedings of the Fifth Berkeley Symposium on Mathematical Statistics and Probability, Volume 1: Statistics.* 281-297 (University of California Press).

6 Rauniyar, N., Gupta, V., Balch, W. E. & Yates, J. R., 3rd. Quantitative proteomic profiling reveals differentially regulated proteins in cystic fibrosis cells. *Journal of proteome research* **13**, 4668-4675, doi:10.1021/pr500370g (2014).

7 Ooshio, T. *et al.* Involvement of LMO7 in the association of two cell-cell adhesion molecules, nectin and E-cadherin, through afadin and alpha-actinin in epithelial cells. *The Journal of biological chemistry* **279**, 31365-31373, doi:10.1074/jbc.M401957200 (2004).

8 Tanaka-Okamoto, M. *et al.* Increased susceptibility to spontaneous lung cancer in mice lacking LIM-domain only 7. *Cancer Sci* **100**, 608-616, doi:10.1111/j.1349-7006.2009.01091.x (2009).

9 Pankow, S. *et al.* F508 CFTR interactome remodelling promotes rescue of cystic fibrosis. *Nature*, doi:10.1038/nature15729 (2015).

10 Fu, L. *et al.* The R345W mutation in EFEMP1 is pathogenic and causes AMD-like deposits in mice. *Hum Mol Genet* **16**, 2411-2422, doi:10.1093/hmg/ddm198 (2007).

11 McLaughlin, P. J. *et al.* Lack of fibulin-3 causes early aging and herniation, but not macular degeneration in mice. *Hum Mol Genet* **16**, 3059-3070, doi:10.1093/hmg/ddm264 (2007).

12 Fernandez-Godino, R., Garland, D. L. & Pierce, E. A. A local complement response by RPE causes early-stage macular degeneration. *Hum Mol Genet* **24**, 5555-5569, doi:10.1093/hmg/ddv287 (2015).

13 Hulleman, J. D., Balch, W. E. & Kelly, J. W. Translational attenuation differentially alters the fate of disease-associated fibulin proteins. *FASEB J* **26**, 4548-4560, doi:10.1096/fj.11-202861 (2012).

14 Roybal, C. N., Marmorstein, L. Y., Vander Jagt, D. L. & Abcouwer, S. F. Aberrant accumulation of fibulin-3 in the endoplasmic reticulum leads to activation of the unfolded protein response and VEGF expression. *Invest Ophthalmol Vis Sci* **46**, 3973-3979, doi:10.1167/iovs.05-0070 (2005).

15 Hulleman, J. D. & Kelly, J. W. Genetic ablation of N-linked glycosylation reveals two key folding pathways for R345W fibulin-3, a secreted protein associated with retinal degeneration. *FASEB J* **29**, 565-575, doi:10.1096/fj.14-255414 (2015).

16 Park, S. K., Venable, J. D., Xu, T. & Yates, J. R., 3rd. A quantitative analysis software tool for mass spectrometry-based proteomics. *Nature methods* **5**, 319-322, doi:10.1038/nmeth.1195 (2008).
